# Unusual complications following left ventricular assisted device implantation: case series

**DOI:** 10.1186/s13019-021-01445-7

**Published:** 2021-04-06

**Authors:** Amjad Shalabi, Erez Kachel, Yigal Kassif, Muin Faqeeh, Preisman Sergey, Leonid Sternik, Liza Grosman-Rimon, Wadi Kinany, Offer Amir, Eylon Ram, Jacob Lavee, Avishay Grupper

**Affiliations:** 1 Department of Cardiac Surgery, Chaim Sheba Medical Center, Tel-Hashomer, 52621 Ramat-Gan, Israel; 2 Affiliated to Sackler Faculty of Medicine, Tel Aviv University, Tel Aviv, Israel; 3 Cardiovascular Department and Research Center, Poriya Medical Center, Tiberias, Israel; 4 Affiliated to the Azrieli Faculty of Medicine, Bar-Ilan University, Safed, Israel; 5 Department of Anesthesia, Chaim Sheba Medical Center, Tel-Hashomer, 52621 Ramat-Gan, Israel

**Keywords:** Left ventricular assisted device, Myoma, Cable damage, Display failure

## Abstract

**Background:**

While left ventricular assisted devices (LVAD) have revolutionized the treatment of advanced heart failure, they are associated with a wide range of complications, including bleeding and infection which are the most common complications reported in the literature. Our case series report four unusual complications not related to gastrointestinal bleeding and infections and their management.

**Case presentation:**

A 61 year old female after LVAD implantation with late onset of severe symptomatic aortic regurgitation treated by transfemoral transcatheter valve implantation (TAVI) with good long term results.

A 75 year old male patient with acute pump failure secondary to cable damage, who underwent urgent pump replacement. A 49 year old female patient with a history of myoma who developed massive uterine bleeding which was treated with emergent open hysterectomy after failed gonadotropin-releasing hormone therapy replacement. A 57 year old male patient with device display failure 1 month after LVAD implantation without the ability to monitor speed, power consumption and blood flow.

**Conclusions:**

LVAD patients can be presented with a great variety of complications. Physicians should be aware of their manifestations and the management options.

## Background

In light of the ongoing shortage of available organs for heart transplantation, left ventricular assist devices (LVADs) continue to gain popularity and widespread acceptance as effective therapy for advanced heart failure. The LVAD can be utilized as a bridge to heart transplantation or permanent destination therapy [1, 2].

However, LVAD support is associated with a variety of complications. Most published studies have focused on gastrointestinal bleeding (GI) and infections especially driveline infections. GI bleedings complications are usually a manifestation of arteriovenous malformation and angiodysplasia related to the continuous-flow LVAD. LVAD related bleeding complications outside of the GI tract may be attributed to the anticoagulation therapy or acquired von-wileibrand disease. Ischemic and hemorrhagic stroke are associated with poor outcomes. The incidence in the first several months after LVAD implantation approximates 8–25% [3].

Few studies have addressed unusual complications following LVAD implantations together with their therapeutic approaches.

We describe here four unusual cases with neither gastrointestinal bleeding nor infectious complications following LVAD implantation, with an emphasis on patient management.

## Case presentation

We retrospectively ran a database search in order to identify LVAD supported patients who had unusual complications. Between 2008 and 2019, 130 patients were implanted with assist devices. 3 of 73 HeartMate II patients (4%) and 1 of 49 HeartMate III (2%) had unusual complications not related to GI bleeding and infection. Of them, we identified one patient with late symptomatic severe aortic regurgitation; one patient with massive uterine bleeding due to myoma, who was treated with emergent open hysterectomy; one patient with acute pump failure secondary to cable damage treated with urgent pump implantation, and an additional patient with display failure following HeartMate III implantation. Table 1 summarizes the hemodynamic parameters of the patients prior to LVAD implantation.

**Table 1:** Baseline Characteristics

	Case A patient	Case B patient	Case C patient	Case D patient
Age (years)	61	75	49	57
Gender	Female	Male	Female	Male
Etiology of cardiomyopathy	Non-ischemic	Ischemic	Non-ischemic	ischemic
LVEF(%)	15	20	10	12
LVEDD (mm)	82	76	78	80
Cardiac Index (l/min/m^2^)	1.9	1.7	2	1.9
PCWP (mmHg)	24	19	22	20
PAPs (mmHg)	32	37	40	38
PAPm (mmHg)	20	25	30	26
CVP (mmHg)	6	8	6	5
NYHA functional class	IV	IV	IV	IV

*LVEF* Left ventricular ejection fraction. *LVEDD* Left ventricular end diastolic diameter; *PCWP* Pulmonary capillary wedge pressure; *PAP* Pulmonary arterial pressure; *CVP* Central venous pressure; *NYHA* New York Heart Association

### Case 1

A 61-year-old female patient with severe idiopathic dilated cardiomyopathy underwent HeartMate II LVAD (Abbott Laboratories, Abbott Park, IL, USA) implantation in November 2010 as a bridge to a heart transplant. The patient gradually developed symptoms of severe left heart failure, and echocardiography assessment showed severe non-calcified aortic valve regurgitation (AR). Our local heart team recommended transfemoral transcatheter aortic valve implantation (TAVI) as the treatment of choice.

After discussing all possible options with the patient, she gave her consent to undergo the procedure. In August 2012 the procedure was performed under mild sedation without reduction or interruption of the LVAD rotation speed (9400 rpm). The right femoral artery was used for access after local anesthesia, and an 18-french guiding sheath was introduced percutaneously with no pre-dilation of the aortic valve. A stiff guide-wire was placed in the left ventricle, carefully avoiding entrapment in the LVAD apical inflow cannula. A 29 mm core valve (Medtronic, Minneapolis, MN, USA) was implanted without need for rapid pacing. Aortography showed minimal residual AR and no blockage of the LVAD outflow graft was seen in the ascending aorta. The patient was hemodynamically stable throughout the procedure and recovered without complications. Echo on the second post-TAVI day showed no residual AR and she was discharged from hospital. An echo test after 56 months showed normal valve function with no aortic regurgitation. This is considered to be one of the longest long term follow up in the literature in patients after LVAD transplantation. However, due to recurrent bleeding and multiple blood transfusions, she developed antibodies and was therefore removed from the heart transplantation list. The patient died in August 2017 due to an ischemic cerebrovascular accident.

### Case 2

**Fig. 1:**
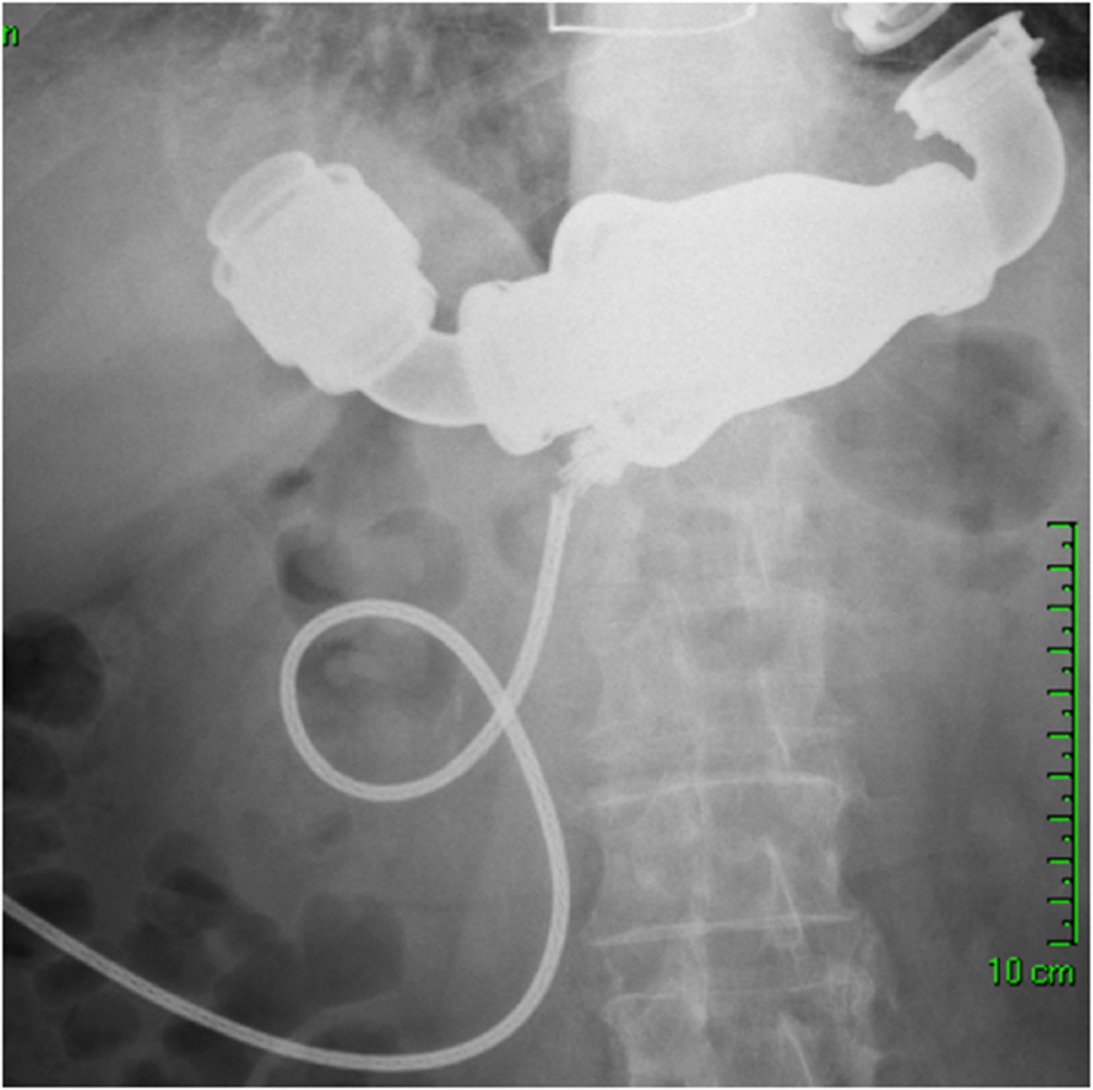
Cable damage after LVAD Implantation

A 75-year-old male patient with severe ischemic cardiomyopathy underwent implantation of HearMate II LVAD and tricuspid valve annuloplasty in December 2013. In April 2014, he was admitted to the emergency room with general weakness, abdominal pain and a red heart alarm in the LVAD system controller. Chest and abdomen X-rays showed a partial disconnection of the driveline from the pump (Fig.1). During preparation for surgery, the LVAD ceased to function and it was decided that the patient undergo urgent pump replacement. There were no operative complications. On post-operative day 3, the patient underwent extubation, and on day 7 in the early hours of the night, the patient died from acute respiratory failure due to aspiration pneumonia. Prior to his death, the LVAD was functioning normally.

### Case 3

A 49-year-old female patient with a history of uterine myoma was admitted to our medical center in October 2015 because of non-ischemic dilated cardiomyopathy, with severely reduced left ventricular function (ejection fraction = 10%), moderately reduced right ventricular function, and severe tricuspid regurgitation. She underwent transplantation with a HeartMate II LVAD and annuloplasty of the tricuspid valve on October 2015. The post-operative period was uneventful, with an increase in her cardiac output to approximately 4 L/min following the LVAD implantation. She was discharged on post-operative day 9, but was re-admitted to the emergency department with excessive vaginal bleeding, weakness and dizziness 1 week after discharge. The patient was put on an anti-coagulation regime when the bleeding started; her international normalized ration (INR) level was 3.1. We started gonadotropin-releasing hormone (GnRH) treatment and, in addition, she underwent pelvic angiography, which was unable to determine the source of the bleeding. While under GnRH treatment, the bleeding ceased and the patient was discharged. However, 2 weeks later, she was re-admitted with massive vaginal bleeding, her INR level was 2.8, and her hemoglobin level was 6.2 g/dl. Vaginal ultrasonography demonstrated coagulum in the uterine cavity. Her clinical condition did not improve after blood product transfusions and her hemoglobin continued to drop. At a multi-disciplinary team meeting, it was agreed to perform emergent open hysterectomy. The patient recovered well after the operation without complications. She was discharged on day 5 with INR at 2.4. The patient remained on long-term aspirin and warfarin therapy with therapeutic INR levels and no vaginal bleeding until she underwent a heart transplant in August 2018. Her post-transplant course was uneventful.

### Case 4

A 57-year-old male patient underwent HeartMate III (Abbott, Chicago, IL, USA) LVAD implantation in December 2016 due to severe ischemic cardiomyopathy. He was the first patient in Israel to receive the HarteMate III LVAD. The operative course was uneventful. Extubation was done on post-operative day 1, and the patient was discharged on day 10. One month after the implantation, controller malfunction appeared, and the numbers disappeared from the controller screen. Even after connecting the patient to an external screen, data regarding pump speed, power and flow were unobtainable. The heart team was required to deal with the dilemma of whether to re-implant a LVAD or to follow up the patient clinically. Experts from the LVAD company who came from abroad were unable to solve the problem. We decided to place the patient at the top of the waiting list for a heart transplant. In **November 2017**, the patient underwent heart transplantation. The post-transplant course was uneventful.

## Discussion and conclusions

As the number of LVAD-supported patients continues to increase, treatment of unusual complications emanating from LVAD implantations becomes more essential. We present here four cases of patients with rare complications following LVAD implantation and their management.

One of the complications of prolonged use of LVAD is the development and progression of de novo aortic regurgitation [4]. Significant AR reduces LVAD output and leads to end-organ mal-perfusion and recurrence of heart failure symptoms. Furthermore, AR affects patient survival [5]. Treatment of late symptomatic AR in LVAD-supported patients represents a real challenge. Surgical aortic valve replacement is associated with increased mortality. While percutaneous aortic valve closure with an Amplatzer occlude device has been reported, significant residual regurgitation has been observed [6]. TAVI poses a potential therapeutic option for patients with late AR development following LVAD implantation. One reported case from Germany demonstrated the feasibility of transapical TAVI treatment in patients with severe AR after LVAD implantation [7]. Our group reported the first in-human transfemoral TAVI for severe AR in a patient with LVAD [8]. We report here the long-term echocardiographic results following transfemoral TAVI in LVAD-supported patients. Our patient underwent TAVI on August 8, 2012. The last echocardiography, performed 56 months after the procedure, showed a well-functioning bio-prosthetic aortic valve without any valvular insufficiency.

Cable damage associated with pump failure is one of the most severe complications after LVAD implantation. This malfunction usually appears at the proximal portion close to the pump body [9]. While an accidental powerful pull on the cable, weight gain and active lifestyle, all reflect improvement of the patient’s clinical condition, it could also be responsible for lead damage. Recently, due to the improvements of the driveline design, the incidence of cable damage has been significantly reduced. Moazem et al. reported a decrease in the incidence of cable damage from 4.2 to 1.2% after modification of the lead design [10]. Patrov et al. reported no case of cable damage after the design improvement in 2012 [9]. Our patient underwent LVAD implantation in December 2013 after the design modification. In our case, the alarm and X-ray allowed for fast diagnosis of the cable damage. Transthoracic echocardiography excluded cannula malposition, significant aortic valve disease and thrombus in the left ventricle. Case 2 patient underwent an uneventful urgent exchange of the main pump body with the remaining inflow and outflow conduits. It is our routine to perform redo median sternotomy in redo cases. Subcostal approach could be associated with less surgical field exposure increased the risk of air embolism due to the lack of access to the outflow graft and aorta. There were no intraoperative complications. Despite the well-functioning LVAD, the patient died on post-operative day 7 due to respiratory failure. In non-emergent cases, pump replacement can be performed safely with operative mortality < 7% [9]. C-shaped tunneling and cable looping of the driveline prevent accidental cable pulling and damage. Transcutaneous energy sources, which are in advanced stages of development, may solve the problem of cable damage completely in the future. Our group together with others performed the first in-human LVAD implantation using wireless energy transfer technology [11].

Uterine myomas are the most common benign uterine tumors that occur in 20–25% of women at the reproductive age [12]. However, they rarely cause acute complications, such as acute vaginal or intra-peritoneal hemorrhage, which can be associated with significant morbidity and mortality. Anticoagulation, which is mandatory for preventing pump thrombosis, may increase the risk of uterine bleeding [13].

Case **3** patient was admitted 15 days after discharge with massive vaginal bleeding. We started treatment with GnRH analogues, but despite performing a pelvic angiography, we could not identify the source of the bleeding. While GnRH has been used recently to achieve hypoestrogenism both as primary conservative therapy for myomas and as an adjunct to myomectomy, its effects are transient, and the myomas usually return to pre-therapy size within a few months of discontinuation [14]. The bleeding ceased and our patient was discharged. Two weeks later, she was re-admitted in stable condition with massive vaginal bleeding. She underwent eventless emergent open hysterectomy after the correction of INR. Laparoscopic hysterectomy was not an option due to the emergency of the case and the reduced function of the right ventricle. During laparoscopic surgery, pneumoperitoneum is necessary to allow adequate exposure. Pneumoperitoneum reduces cardiac output due to direct compression to the inferior vena cava causing reduced preload [15]. Urine artery embolization has been used for the treatment of hemorrhaging that complicates the course of uterine myoma. However, the procedure is compromised by the high rates of post-procedural complications and high rates (28–32%) of re-intervention [16].There were no complications following hysterectomy and the patient was discharged 5 days after the procedure. The patient was 2.5 years without vaginal bleeding, during which time she was under treatment with aspirin and Coumadin maintaining a therapeutic level of INR. Heart transplant was performed in August 2018, and her post-transplant course was uneventful.

While today most LVAD patients are managed as outpatients, their follow-up is complex. In addition to INR management, it is also necessary to monitor several flow parameters of the device. Monitoring of the speed, power consumption and blood flow is mandatory. Any changes in these three key parameters will alert both the patient and the physician to abnormalities in the LVAD function. Our patient (Case **4**) had display failure 1 month after LVAD implantation. Even after connecting the patient to an external screen, we were unable to receive information about pump speed, power consumption and blood flow. Experts from the company were unable to solve this rare problem. Device replacement is not an entirely harmless procedure and is associated with increased risk of morbidity and mortality [9]. Since our patient was asymptomatic we decided to continue follow-up clinically echocardiographically. We monitored the flow by Doppler ultrasound. In selected cases swan ganz catheter can be used. We placed the patient at the top of the waiting list for a heart transplant. He was discharged after 1 week of observation in our department, and asked to be in close contact with us. He monitored his symptoms, weight and edema daily, and after 11 months underwent heart transplantation. The post-transplant course was uneventful.

According to the company, the reason for the display failure was due to a communication error between the patient’s system controller and the LVAD pump. It triggered a communication fault advisory alarm on the system controller. The company associated the error to manufacturing variances from a single supplier that could have led to crystallization formation, which may then have disrupted communication between the pump and the system controller. The company has updated the specification and manufacturing process to ensure that this error does not re-occur during HeartMate III device manufacturing.

## Data Availability

Not applicable.

## Abbreviations

LVAD: Left ventricular assisted device
TAVI: Transcatheter valve implantation
GI: Gastrointestinal bleeding
AR: Aortic valve regurgitation
INR: International normalized ration
GnRH: Gonadotropin-releasing hormone
