# Correction to: Unusual complications following left ventricular assisted device implantation: case series

**DOI:** 10.1186/s13019-021-01633-5

**Published:** 2021-09-06

**Authors:** Amjad Shalabi, Erez Kachel, Yigal Kassif, Muin Faqeeh, Preisman Sergey, Leonid Sternik, Liza Grosman-Rimon, Wadi Kinany, Offer Amir, Eilon Ram, Jacob Lavee, Avishay Grupper

**Affiliations:** 1 Department of Cardiac Surgery, Chaim Sheba Medical Center, Tel-Hashomer, 52621 Ramat-Gan Israel; 2 Affiliated to Sackler Faculty of Medicine, Tel Aviv University, Tel Aviv, Israel; 3 Cardiovascular Department and Research Center, Poriya Medical Center, Tiberias, Israel; 4 Affiliated to the Azrieli Faculty of Medicine, Bar-Ilan University, Safed, Israel; 5 Department of Anesthesia, Chaim Sheba Medical Center, Tel-Hashomer, 52621 Ramat-Gan Israel

## Correction to: Journal of Cardiothoracic Surgery (2021) 16:70 10.1186/s13019-021-01445-7

The original article [1] contained an error in co-author, Eilon Ram’s name which has since been corrected.

## References

[1] Shalabi A (2021). Unusual complications following left ventricular assisted device implantation: case series. J Cardiothorac Surg.

